# Undifferentiated Pleomorphic Sarcoma of the Hand with Lung Metastases

**DOI:** 10.7759/cureus.5557

**Published:** 2019-09-03

**Authors:** Emily Whiles, Hareesh Joshi, Olukunle Jenyo, Arun Perumalthiagarajan, Samson O Oyibo

**Affiliations:** 1 General Surgery, Peterborough City Hospital, Peterborough, GBR; 2 Internal Medicine, Peterborough City Hospital, Peterborough, GBR; 3 Diabetes and Endocrinology, Peterborough City Hospital, Peterbourough, GBR; 4 Diabetes and Endocrinology, Peterborough City Hospital, Peterborough, GBR

**Keywords:** undifferentiated pleomorphic sarcoma, hand, metastases

## Abstract

Undifferentiated pleomorphic sarcoma (UPS) is a rare tumor of mesenchymal origin affecting the hand in less than 3% of cases. A 92-year-old male reported an enlarging, painless mass on his left hand of three months duration. Examination of the hand revealed a soft, non-tender, immobile mass over the wrist joint, just proximal to his thumb. Immunohistochemistry findings on ultrasound-guided biopsy were consistent with UPS. Further intractable pain, ulceration, and bleeding necessitated urgent radiotherapy, which provided no relief. This was followed by amputation above the elbow. Seven months later, the patient presented with confusion. A chest X-ray revealed extensive bilateral pulmonary metastases. In light of this result, the patient was referred to the palliative care team. UPS carries a poor prognosis, with a high risk of metastases. Early diagnosis and treatment is required for optimal clinical outcomes. Through this case, we highlight the need for a more effective treatment strategy to improve clinical outcomes.

## Introduction

Sarcomas are a heterogeneous group of rare malignant mesenchymal tumors, which can be categorized into either bone or soft tissue sarcomas. They have an annual incidence of two to three cases per 100,000 individuals [1]. Although these can occur throughout the body, only 15% occur in the upper extremity and less than 3% of all soft tissue sarcomas present in the hand [2]. We present a case of undifferentiated pleomorphic sarcoma (UPS) of the hand in a patient who subsequently developed pulmonary metastases despite having neoadjuvant radiotherapy and radical amputation.

## Case presentation

**Figure 1:**
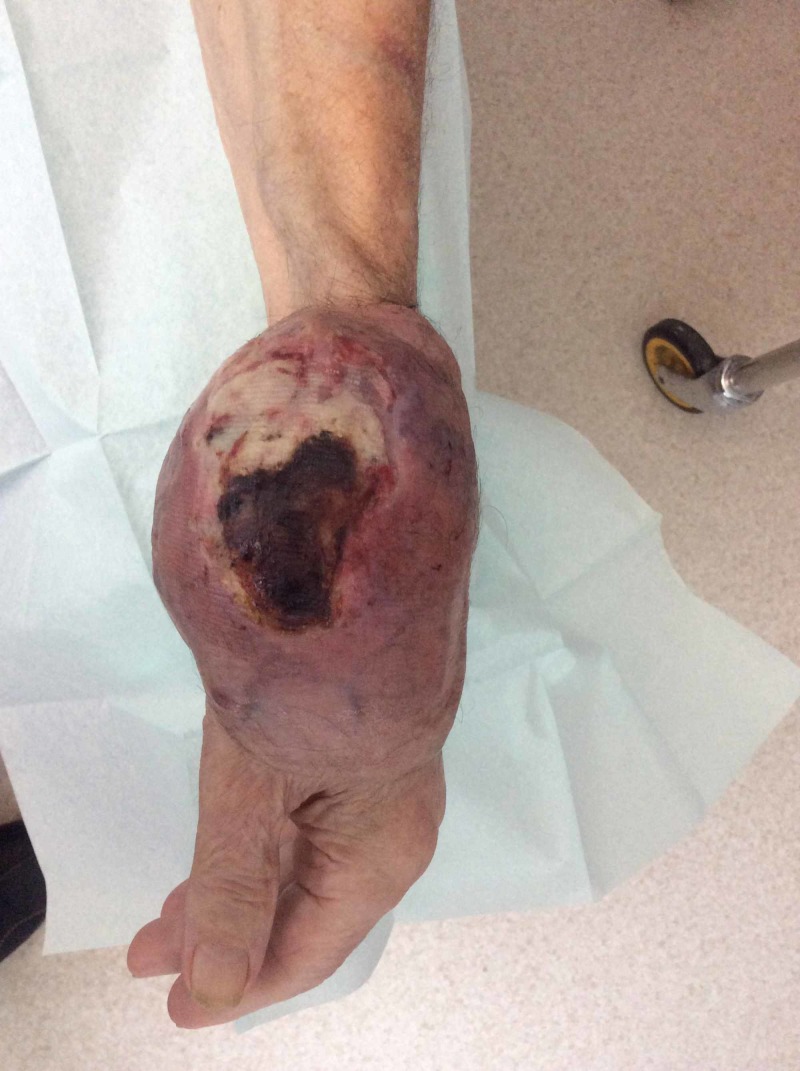
Left hand Large tumor over the wrist joint with ulceration and bleeding (15 cm x 10 cm)

A 92-year-old, right-handed male presented to the oncology department with a three-month history of a rapidly enlarging, painless mass on the dorsum of his left hand. He had a past medical history of type 2 diabetes mellitus, hypertension, atrial fibrillation, and cerebral vascular disease. On examination of the hand, there was a 7 cm x 6 cm soft, non-tender, and immobile mass on the lateral aspect of the left hand, with no tethering to the overlying skin. There was a full range of movement of his wrist joint and fingers, and there were no neurological deficits. Repeat examination a few months later revealed that the mass had increased in size, ulcerated, and began to bleed (Figure 1).

An initial ultrasound scan demonstrated a large mass arising from the radial wrist, measuring approximately 6.4 cm x 5.3 cm x 2.7 cm. Before a biopsy was attempted, a computed tomography (CT) angiogram was performed to rule out any aneurysm within the mass. This demonstrated that the mass had increased in size (diameter of 8.5 cm) after two months. An ultrasound-guided biopsy of the hand mass was performed: this displayed moderately pleomorphic epithelioid spindle cells within a myxoid stroma containing thin-walled and compressed vessels. These features were suggestive of a spindle cell sarcoma.

A CT scan performed two months later demonstrated a mass lesion measuring 12.5 cm x 8 cm x 6 cm, centered around the level of the radiocarpal joint and enveloped the radial artery. A staging CT scan of the chest, abdomen, and pelvis revealed a single 4-mm nodule in the upper lobe of the right lung. This was thought to be a simple nodule, and, therefore, a surveillance CT scan of the chest was booked to take place in four months.

**Figure 2:**
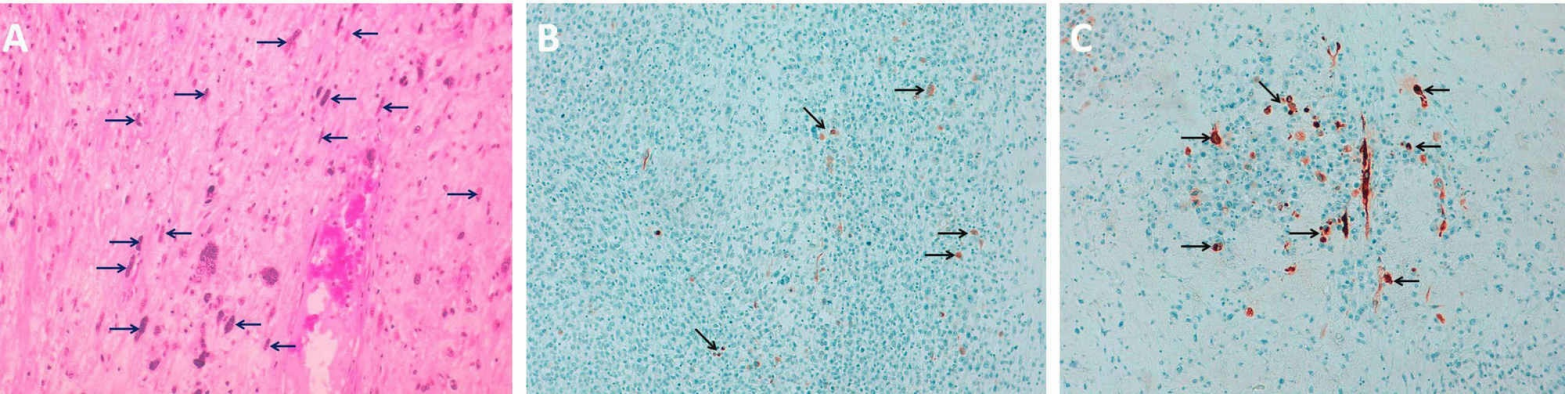
Immunohistochemical study A. Hematoxylin and eosin staining showing multiple spindle cells (arrows), B. Scanty staining for cytokeratin AE1/AE3 (arrows) and C. Patchy positive staining for desmin (arrows).

Because of extreme pain and bleeding from the tumor, neoadjuvant radiotherapy was given to reduce tumor size and relieve pain. However, despite the radiotherapy, there was no appreciable improvement in size or symptoms. After extensive multidisciplinary team discussions, a left above-the-elbow amputation with wide safety margins was performed to provide the patient with a better quality of life. Histopathology examination of the post-surgical specimen illustrated ovoid-spindle cells and pleomorphic tumor cells with no definite line of differentiation (mitotic count more than 20 per 10 high-power fields in some areas). The immunohistochemical examination demonstrated negative staining for S-100 and CD34, scanty staining for cytokeratin AE1/AE3 and patchy positive staining for desmin. These features confirmed a high-grade (grade 3) UPS (Figure 2).

**Figure 3:**
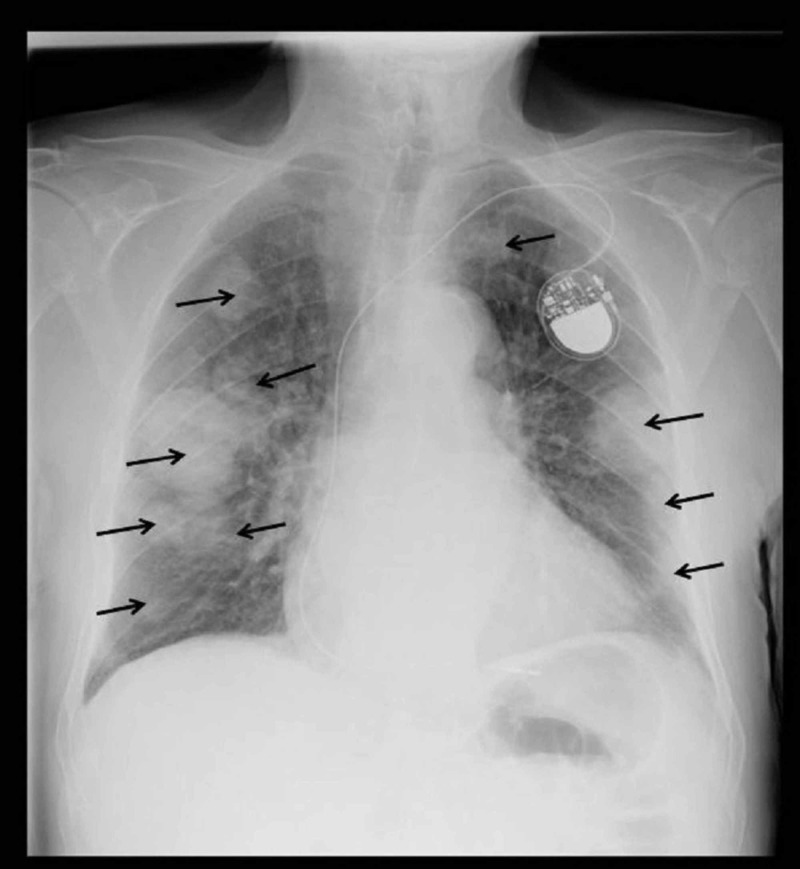
Chest X-ray Showing large bilateral lung field metastases (black arrows)

Seven months later, the patient presented to the emergency department with a productive cough and confusion. A chest X-ray revealed extensive bilateral pulmonary metastases (Figure 3). In view of these findings, a palliative approach was deemed appropriate and a referral was made to the Macmillan Cancer Support Team.

## Discussion

Undifferentiated pleomorphic sarcoma (UPS) is an aggressive malignant tumor affecting the soft tissues. UPS has no specific cell of origin or histiocytic line of differentiation and is usually a diagnosis of exclusion [3]. It commonly occurs in adults aged 60-70 years and presents as a painless, rapidly enlarging nodule affecting the lower limbs [4].

Histology is essential in determining the diagnosis; characteristic features include a combination of storiform and pleomorphic areas containing pleomorphic spindle cells with marked atypia, numerous mitotic figures, and areas of necrosis with lymphohistiocytic infiltrates [5]. When UPS occurs in the skin, it can be difficult to differentiate it from other skin sarcomas, however, skin sarcomas have strong positive staining for cytokeratin AE1/AE3. Other differential diagnoses, such as rhabdomyosarcomas and leiomyosarcomas have strong positive staining for desmin. On the other hand, UPS usually exhibit negative staining for cytokeratin AE1/AE3, S-100, and CD34 and are negative to occasional patchy staining for desmin.

Treatment involves total surgical excision of the tumor with wide safety margins preceded by adjuvant radiotherapy in patients with non-metastatic UPS; however, the high-grade nature of the tumor limits any benefit from localized treatments.

UPS has a significant recurrence rate with 44% of patients experiencing local invasion and 42% metastasizing despite surgical excision of the primary tumor [4]. Risk factors influencing the survival, metastatic, and local recurrence rates include histological grade as well as tumor size and depth [4]; Weiss and Enzinger analyzed 200 cases of UPS and found that tumors less than 2.5 cm in size had a metastatic rate of 21% as compared with a rate of 57% in those greater than 10 cm in size [4]. The median survival rate for patients with UPS is 65%-70% over five years; however, recurrence and distant metastases often develop within 12-24 months of diagnosis, commonly occurring in the lung (90%), bone (8%), and liver (1%) [5].

Although intensive chemotherapy and the establishment of surgical procedures have improved the outcomes of patients with sarcoma, the curative rate of recurrent and metastatic sarcomas is still not satisfactory. A previous study compared standard neoadjuvant chemotherapy (epirubicin and ifosfamide) with histotype-tailored neoadjuvant chemotherapy (gemcitabine and docetaxel) for the treatment of UPS. This study did not demonstrate any superiority between the two regimens, although the common side effects of thrombocytopenia, anemia, and neutropenia occurred less frequently in the histotype-tailored chemotherapy group [6]. There are currently developing fields of research into target-specific chemotherapy that will affect the mechanisms of progression and metastases of malignancies. However, current studies have not demonstrated superiority over the standard and histotype-tailored therapies for UPS. Further studies are required [7].

This is a unique case in that the age and location of presentation were highly unusual when compared to available case studies. Additionally, the rate of metastases was far faster than the usual 12-24-month time window. It is highly probable that the suspected pulmonary nodule found on the initial CT scan of the chest was the first sign of early metastases.

## Conclusions

Early and accurate histological typing, grading, and staging of UPS is essential in determining the prognosis and subsequent management of patients. There should be a low threshold for radical surgery due to the high local recurrence and metastatic rates. Through this case, we highlight the need for a more effective treatment strategy to improve clinical outcomes.
